# Limits of Coherency and Strain Transfer in Flexible 2D van der Waals Heterostructures: Formation of Strain Solitons and Interlayer Debonding

**DOI:** 10.1038/srep21516

**Published:** 2016-02-12

**Authors:** Hemant Kumar, Liang Dong, Vivek B. Shenoy

**Affiliations:** 1 Department of Materials Science and Engineering, University of Pennsylvania, Philadelphia, 19104, USA

## Abstract

In flexible 2D-devices, strain transfer between different van-der Waals stacked layers is expected to play an important role in determining their optoelectronic performances and mechanical stability. Using a 2D non-linear shear-lag model, we demonstrate that only 1-2% strain can be transferred between adjacent layers of different 2d-materials, depending on the strength of the interlayer vdW interaction and the elastic modulus of the individual layers. Beyond this critical strain, layers begin to slip with respect to each other. We further show that due to the symmetry of the periodic interlayer shear potential, stacked structures form strain solitons with alternating AB/BA or AB/AB stacking which are separated by incommensurate domain walls. The extent and the separation distance of these commensurate domains are found to be determined by the degree of the applied strain, and their magnitudes are calculated for several 2D heterostructures and bilayers including MoS_2_/WS_2_, MoSe_2_/WSe_2_, Graphene/Graphene and MoS2/MoS2 using a multiscale method. As bilayer structures have been shown to exhibit stacking-dependent electronic bandgap and quantum transport properties, the predictions of our study will not only be crucial in determining the mechanical stability of flexible 2D devices but will also help to better understand optoelectronic response of flexible devices.

There has been a growing interest in flexible electronics and devices ranging from flexible displays to electronic skin have already been prototyped^1^,^2^,^3^. The optoelectronic performances of these devices can be greatly enhanced by stacking two or more layers of different 2D materials^4^,^5^,^6^,^7^,^8^. During normal operations of these flexible devices, the 2D heterostructures frequently bear a certain degree of strain, *e.g.,* when a flexible structure onto which the 2D heterostructures are placed is either bent or stretched. Due to the weak van der Waals (vdW) coupling between different layers of the 2D structures^9^, the mechanical stresses in one layer of the heterostructure need not always be completely transferred to the adjacent layers. The layers in stacked 2D heterostructures are expected to remain coupled with each other when the strain in the substrate layer is small, and can potentially become completely decoupled as the strain increases beyond a critical value. There are many open questions related to this phenomenon in 2D heterostructures. For example, how is the strain transferred from one layer spatially distributed in the other layers? When does debonding/sliding between the layers start? What is the magnitude of the critical strain that causes debonding between the adjacent 2D layers as a function of the vdW interaction strengths and other materials properties? Answers to these questions are of paramount importance for the development of flexible devices based on 2D materials because of two reasons. First, debonding between different layers of heterostructures will make flexible devices mechanically unstable. Second, the strain and stacking configurations have a significant impact on the electronic and optoelectronic properties of 2D materials such as the bandgap energy and the carrier mobilities^10^,^11^,^12^,^13^. Hence, it is crucial to understand strain transfer mechanisms between different layers of 2D materials. The purpose of this study is to develop a theoretical model that predicts the distribution of strains in a bilayer 2D heterostructure material with the bottom layer subject to an external load (*e.g.* when it is stretched) while the top layer is free to deform. This model for a bilayer system can easily be generalized for the multilayer systems.

The morphology and deformation patterns for any interface between stacked 2D layers are determined by the competition between the vdW energy and the elastic energy associated with the deformation of individual layers. This competition has led to many interesting observations in 2D materials. For instance, commensurate-incommensurate domains (strain solitons) are formed^14^ in bilayer graphene when the two layers are either translated or rotated by a small amount from the minimum energy Bernal stacking. Similarly, heterostructures of GR-hBN deform to form moiré patterns when the two layers are in perfect alignment, but decouple from each other when the misalignment angle is larger than 10° 15,16. This interplay of energies is the basic premise of the shear-lag model studied here. Linear elastic models have been used to study the strain transfer in multiwall carbon nanotubes^17^ and polymer-composites^18^. However, these linear models cannot predict the formation of strain solitons and thus fail to provide estimates for the critical strain at which interface sliding and debonding occurs.

To address these issues, we have developed and implemented multiscale formulation based on a non-linear shear-lag model to investigate the mechanism of the strain transfer between different layers of 2D materials. The interlayer shear potential takes into account the underlying lattice symmetry and 2D periodicity, and interlayer slip and soliton formation are natural outcomes of our model. For the purpose of demonstration, we choose the recently synthesized vdW heterostructure of MoS_2_/WS_2_ and bilayer graphene. Our model predicts that for MoS_2_/WS_2_, a maximum strain of 1.8% can be transferred from the bottom WS_2_ layer to the top MoS_2_ layer and beyond this strain debonding/slippage between the two layers is observed. For the graphene bilayer the critical strain to begin debonding is 0.53%. Furthermore, we show that strain solitons are formed due to the periodicity of the interlayer shear-potential whose distribution depends on the magnitude of the applied strain. We also show that strain solitons in MoS_2_/WS_2_ have AB stacking, but a graphene bilayer has alternating AB/BA stacked solitons, which is consistent with the recent experimental observations^19^,^20^,^21^ of coexisting AB/BA stacked domains in graphene bilayers. The rest of the paper is organized as follows: first, we describe the 2D shear-lag model wherein one of the layers (bottom) bears a tensile strain and the other layer (top) is free to relax. Next, we discuss the predictions of our model for MoS_2_/WS_2_ heterostructures and graphene bilayers and then explain the origin of the qualitatively distinct behaviors for these two cases based on the features of their interlayer shear potential. To validate our model, we compare our results with an all atom molecular mechanics simulation. Finally we present theoretical predictions for strain transfer and debonding in other vdW heterostructures, namely, MoS_2_/MoS_2_, WS_2_/WS_2_, MoSe_2_/WSe_2_, MoSe_2_/MoSe_2_, and WSe_2_/WSe_2_.

## Method and Details

**Figure 1:**
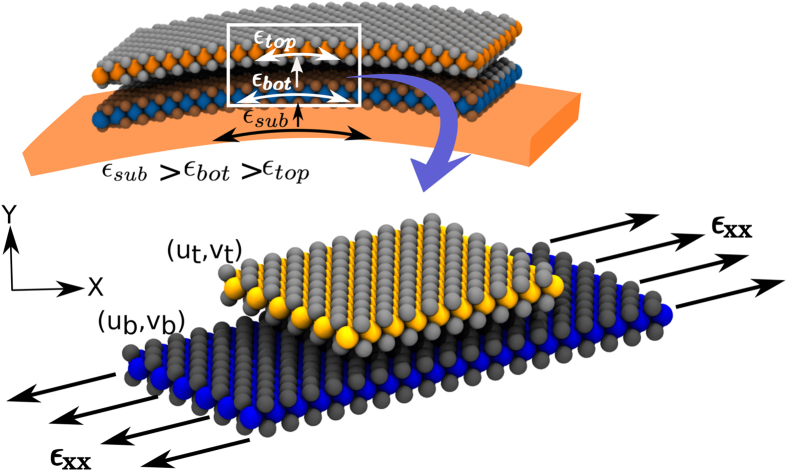
Schematic of the 2D shear-lag model. Top figure represents a typical scenario in experiments, wherein the flexible substrate is subjected to the deformations and subsequent strain-transfer between different layers in the heterostructure will be important. Bottom figure shows the schematic of the model studied here. The bottom layer is subjected to external stresses (as shown by arrows) that introduce strain in the bottom layer and the top layer is free to relax at the edges.

The total energy of the 2D heterostructure (*U*_*tot*_) (Fig. 1) can be written as:


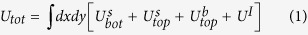


where the superscripts *s, b* and *I* represent the strain energy, the bending energy and the interlayer shear energy, respectively. Subscripts *top* and *bot* in Eq. (1) denote the top and bottom layers, respectively. Each layer is modeled as a thin elastic plate. The strain energy of each of the plates is given as:


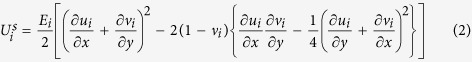


where the subscript ‘*i’* can be either *top* (top layer) (t) or *bot* (bottom layer) (b), and *E*_*i*_, *u*_*i*_ and *v*_*i*_ are the 2D elastic modulus, the displacement along the *x* a*x*is, and the displacement along the *y* axis, respectively, for the *i*^th^ layer. Similarly, bending energy of the top plate is given by:


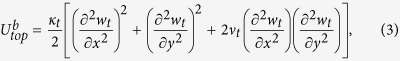


where *k*_*t*_ is the 2D bending modulus of the top plate and *w*_*t*_ is the out-of-plane displacement in the top plate. For simplicity, we assume that the out-of-plane displacements exist only in the top layer.

Owing to weak vdW coupling, the two layers may have different displacements at a given point, creating a shift in the stacking configuration given by:


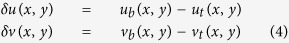


The interlayer energy, which is defined as the vdW energy associated with the shift in the stacking configuration, is given by:


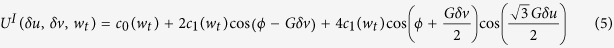


In our study, *U*^*I*^ is a hexagonally symmetric function due to the lattice symmetry of the 2D materials of interest and is characterized by the separation-dependent interlayer coefficients *c*_0_(*w*_*t*_)*,c*_1_(*w*_*t*_), *ϕ* and reciprocal lattice vector G, for a given heterostructure. These coefficients were obtained using *ab-initio* DFT-TS calculations, which are detailed in the SI. Features of the interlayer potential play an important role in determining the nature of the strain-transfer.

## Results and Discussion

### Depending on the Symmetry of the Interlayer Shear Potential Different Pathways Are Preferred to Relax Interlayer Shear

**Figure 2:**
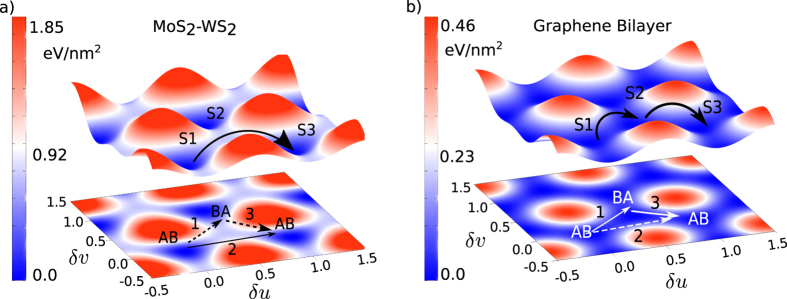
Potential energy surfaces (PES) for the interlayer displacements. (**a**) For a MoS_2_/WS_2_ heterostructure, the original AB stacking (S1 and S3) has a lower energy as compared to the BA stacking (S2). Hence, the transition from S1 to S3 is preferred via Path 2. (**b**) However, for a graphene bilayer S1, S2, and S3 have the same energy. The energy barrier from S1 to S2 is lower than that from S1 to S3. Hence, Path 1 is preferred as compared to Path 2 for interlayer shear relaxation. This pathway leads to a change in stacking configuration from AB to BA and is accompanied by the additional transverse shear of 0.7 Å.

Heterostructures with a lattice mismatch exhibit a shear potential with moiré patterns whose periodicity is determined by the lattice mismatch^15^. However, heterostructures without any lattice mismatch have an interlayer shear potential whose periodicity is identical to the lattice periodicity. Three different high-symmetry configurations can be obtained by displacing one layer relative to the other layer. Based on the energetics of these configurations, two distinct kinds of interlayer shear potential energy surfaces (PES) are possible, as plotted for the case of MoS_2_/WS_2_ heterostructure and bilayer graphene (BLG) in Fig. 2. The maxima (in red) and minima (in blue) in the PES correspond to the energetically most unfavorable and favorable stacking configurations, respectively. If a bilayer structure is originally stacked with the AB sequence (S1 in Fig. 2) and strain is applied along the zigzag direction (*x* axis), two kinds of energy minima are accessible (S2 and S3 in Fig. 2) in the vicinity. S2 corresponds to the BA stacking configuration that is realized by an interlayer shift of *a*/2 for the top layer along the armchair direction where ‘*a*’ is the lattice constant. S3 has the same AB stacking configuration as S1 and it is realized by an interlayer shear equivalent to *a*.

For structures like BLG, the AB (S3) and BA (S2) stacking configurations are energetically equivalent. The transition from S1 to S2 (Path 1) can be realized with a smaller interlayer shear (*a*/2 vs. *a*) as compared to the transition from S1 to S3 (Path 2) and hence, Path 1 is preferred to relax interlayer shear. On the other hand, for heterostructures like MoS_2_/WS_2_, the BA stacking (S2) has a higher energy as compared to the AB stacking (S3) and hence S2 is not an energetically preferred configuration for such heterostructures and this stacking is not realized. For such heterostructures, Path 2 is preferred to relax interlayer shear.

In the next section, we study the strain transfer in a bilayer MoS_2_/WS_2_ heterostructure (top layer: MoS_2_, bottom layer: WS_2_). The lateral size of the heterostructure is 200 nm by 100 nm and equilibrium structure was assumed to have an AB stacking which is the lowest energy stacking for this construct. The bottom layer is stretched along the zigzag direction (*x* axis) while the top layer is stress free at the edges (see Fig. 1).

### Induced strain in the top layer increases linearly with increasing applied strains and then decreases when the applied strain reaches a critical value

**Figure 3:**
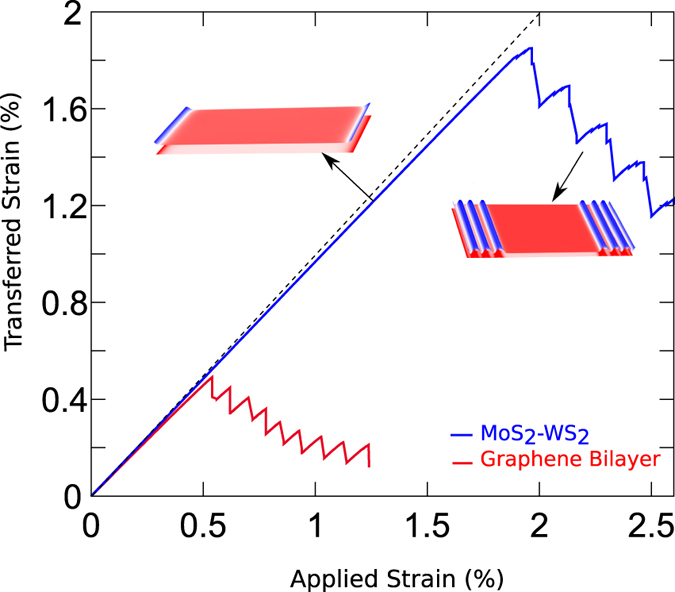
Average strain in the top layer as a function of the applied strain in the bottom layer for the MoS_2_-WS_2_ bilayer and a graphene bilayer. Initially both layers are commensurate with each other and the strain transferred to the top layer increases linearly with applied strain. The dotted line represents expected value of the strain in top layer for the perfectly coupled case. Actual strain is smaller due to the existence of shear lag domains at the edges. Beyond the critical applied strain of 1.8% (0.53% for graphene), incommensurate domains appear and hence the average strain in the top layer starts decreasing with increasing strain. Each dip for the applied strain >1.8% (0.5% for graphene) corresponds to the formation of an incommensurate domain.

To study strain transfer, we compute the average strain in the top layer for different values of the applied strain along the *x* axis. Results obtained from our shear-lag model are plotted in Fig. 3. The average strain in the top layer increases linearly with increasing applied strain until it reaches 1.8% (Fig. 3). However, beyond this critical strain, the average strain in top layer abruptly drops to a much smaller value, indicating the slippage between the two layers. Further increment in the applied strain gradually increases the strain in the top layer until another abrupt drop is observed. This behavior leads to a saw-tooth wave-like strain transfer curve with an overall downwards trend as shown in Fig. 3. This peculiar trend in the strain-transfer curve is a direct consequence of the 2D periodicity of interlayer shear potential and can be understood by studying the spatial distribution of the strain in the two layers.

### Origin of shear-lag domains and undulation in morphology; Decay in the induced strain is caused by the formation of incommensurate domains

**Figure 4:**
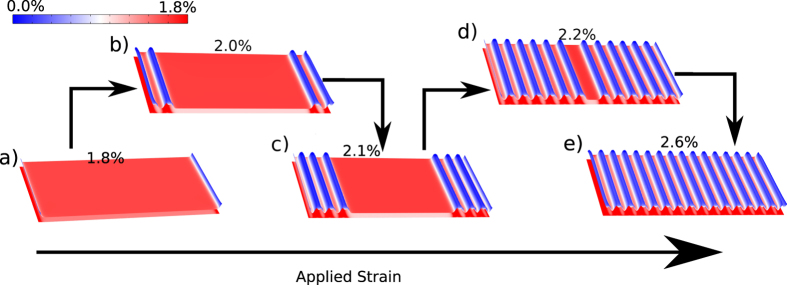
Elastic deformations in both layers for MoS_2_/WS_2_ heterostructure for different values of applied strain, computed from the 2D non-linear shear-lag model. The strain component along the applied strain direction is color coded for both layers. (**a**) Under an applied strain up to 1.8%, both layers have the same strain everywhere in the interior except near the edges (shown in blue), which are incommensurate due to the shear-lag effect. (**b**–**e**) The number of incommensurate domains begins to increase with increasing applied strains. Each incommensurate domain has a smaller strain as compared to the commensurate domain and hence total strain in the top layer decreases with the increasing strain.

When the bottom layer is stretched with respect to the top layer (see Fig. 1), an interlayer shear stress develops between the two layers. To minimize this interlayer shear, atoms of the top layer move with the bottom layer and strain is transferred from the bottom layer to the top layer. Surface plots of the strain distribution in the two layers along with the out-of-plane displacement in the top layer for different values of the applied strain are shown in Fig. 4. For the relatively small strains (less than 1.8%), both layers are commensurate with each other and have the same spatial distribution of strain except for regions near the free edges (Fig. 4). In this strain range, the average strain in the top layer almost equals the applied strain, and the small difference between (Fig. 3) them comes from to the shear-lag domains near the free edges which carry a very small strain in the top layer (Fig. 4). For the strains larger than 1.8%, the cost of the elastic energy to maintain the commensurate state in the entire construct becomes larger than the gain in the vdW energy and creates regions with incommensuration (debonded domains), where the interlayer shear is significant and the tensile strain is relatively small in the top layer (Fig. 4).

In an incommensurate domain, the interlayer-shear decays over a characteristic length, known as shear-lag length *λ* that is determined by the relative magnitude of Young’s Modulus and the coefficients of the interlayer shear potential. For the MoS_2_/WS_2_ heterostructure this length is close to 2.48 nm (Fig. 3). Owing to incompatible registry between the two lattices, the regions with non-zero interlayer shear have a higher vdW energy and experience large out-of-plane forces, leading to undulated morphology in the top layer. The magnitude of the out-of-plane displacements is determined by the competition between the gain in the vdW energy and the cost of the bending energy.

### Strain-transfer in bilayer Graphene

**Figure 5:**
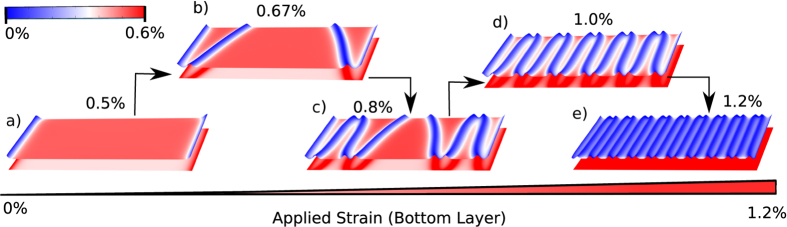
Elastic deformations in the two layers for a graphene bilayer for different values of applied strain. Shape and stacking configuration of commensurate domains are different from MoS_2_/WS_2_ heterostructure, due to equivalence of AB/BA stacking configuration in graphene.

For graphene bilayers, the general trend of strain transfer is similar to that of a MoS_2_/WS_2_ heterostructure. Structural evolution with increasing strains in the bottom layer is shown in Fig. 5. Shear-lag length of this structure is close to 4.14 nm and the graphene bilayer remains fully coupled till 0.53% of applied strain. Beyond this critical strain, the top layer starts to slip and the number of the incommensurate domains increases (Fig. 5). At an applied strain of ~1.4%, the strain transferred to the top layer is significantly small and the two layers are almost debonded (Fig. 3). Although the interlayer interaction in a graphene bilayer is similar to the weak vdW interaction between MoS_2_ and WS_2_ layers, its critical strain for slippage is much smaller. In addition, the stacking configuration of the commensurate domains also differs in the two bilayer structures. For a MoS_2_/WS_2_ heterostructure, all commensurate domains maintain the original AB stacking while the commensurate domains in a graphene bilayer have alternating AB and BA stacking. These two differences can be explained using the features of the interlayer PES as detailed below.

### Strain solitons in bilayer graphene display alternating AB/BA stacking while MoS_2_/WS_2_ heterostructure shows only AB stacking

**Figure 6:**
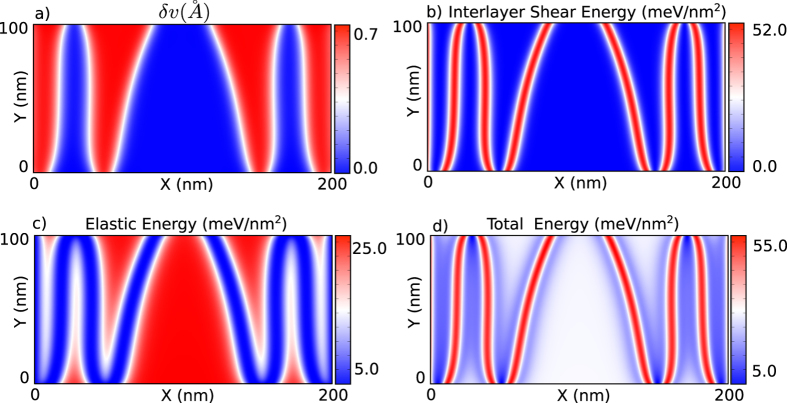
Energetics of the incommensurate-commensurate domains in a graphene bilayer. (**a**) Component of the relative displacements along the armchair (Y) direction when a strain is applied along the zigzag (X) direction. A displacement of 0.7 Angstrom along the Y direction in commensurate domain implies transition from the AB stacking to the BA stacking. (**b**) Distribution of the interlayer shear-energy when average strain in the bottom layer is 0.8%. Commensurate domains exhibit a relatively lower interlayer energy while incommensurate domains have a higher interlayer energy. (**c**) Elastic energy distribution in the top layer. The commensurate domains have a relatively higher elastic energy as compared to the incommensurate domains. Such an arrangement leads to a configuration where the highest energy density is concentrated in the small incommensurate domains, as shown in (**d**).

As explained in the earlier section, for bilayer graphene the minima (S2 and S3) in PES are in the BA and AB stacking configurations, respectively, which are energetically equivalent (See Fig. 2b). However, the transition from initial stacking configuration S1 to S2 (Path 1) can be realized at a much smaller interlayer shear (and hence at a smaller applied strain) as compared to the one from S1 to S3 (Path 2). Moreover, Path 1 (AB to BA) requires an energy barrier of 5 meV compared to of 20 meV for Path 2 (AB to AB). Therefore, Path 1 is the preferred route, leading to BA-stacked domains in bilayer graphene. Note that this transition also creates an intralayer shear along the transverse direction (Y axis). Based on same considerations, when interlayer shear starts to build up in a BA-stacked domain (S2), it relaxes to the AB-stacked domain (Path 3). Path 3 also relaxes the intralayer shear caused by the transverse interlayer shift during the AB to BA transition as shown in Fig. 2b. Therefore, commensurate domains have alternating AB and BA stacking in graphene bilayer. On the other hand, for the MoS_2_-WS_2_ heterostructure, Path 2 is always the preferred route to relax interlayer shear because the final AB stacking state in Path 2 has a lower energy compared to the final BA stacking in Path 1. Therefore, all commensurate domains are in the AB stacking configuration in the MoS_2_-WS_2_ heterostructure. In other words, bilayer graphene and MoS_2_-WS_2_ heterostructure exhibit different stacking behaviors due to different PES characteristics. The incommensurate domains cost vdW energy but have relatively small tensile strains; the commensurate domains have lower vdW energy as they maintain AB/BA stacking at the cost of elastic deformations (Fig. 6).

It should be noted that the domains with alternating AB/BA stacking configurations have been experimentally observed for bilayer graphene structures grown using the chemical vapor deposition (CVD) method^19^. CVD-grown graphene is expected to bear a certain degree of strain due to a lattice mismatch between graphene layers and the substrate, which is similar to the applied strain in our model. To the best of our knowledge, our model is the first one that explains the energetic origin of this phenomenon because the full 2D periodic interlayer shear potential is incorporated in our model. On the other hand, all previous models failed to explain this phenomenon, since they only consider a 1D lattice potential.

### Critical strain for the slippage depends on the ratio of the effective young’s modulus and the strength of the interlayer shear potential

We have also carried out analytical calculations to obtain more insights into the dependence of the critical strain on mechanical properties of the underlying lattices. The governing equations were solved by performing a variational minimization of the total energy using the Euler-Lagrangian formulation. Bending moduli of the 2D materials is almost 3 orders of magnitude smaller compared to their elastic moduli. Therefore, the energy cost of bending can be neglected to obtain simplified analytical solutions. Details of the method used to obtain the solutions are presented in the SI. The numerical results show that the slippage begins when the interlayer shear at the edge reaches half of the bond length. Using this criterion to obtain the critical strain for slippage (*∈*_*c*_) from analytical solutions, we find:


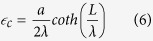


where 
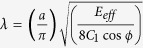
 is the shear-lag length, 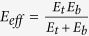 being the effective Young’s Modulus of the bilayer and L is the length of the sheet. It is clear from Eq. 6 that the critical strain is inversely proportional to the shear-lag length, which is smaller for the MoS_2_-WS_2_ heterostructure compared to bilayer graphene. The effective Young’s modulus (*E*_*eff*_) of MoS_2_-WS_2_ heterostructure is smaller and the coefficient of interlayer shear (*c*_*1*_) is larger and hence shear-lag length is much smaller as compared to graphene leading to the higher critical strain of slippage.

### Predictions from our models are in good agreement with all atom molecular simulations and experiments

Next, to validate our method we compare our results with experimental observations. A recent experimental study^18^ observed that a graphene bilayer has a uniform strain distribution when the applied strain is smaller than 0.4% but strain distribution becomes non-uniform for the larger applied strains, validating domain formation predicted by our model. In addition, it was also observed that maximum strain in the bilayer graphene was 0.4%, which is close to the 0.48% calculated by our model. We further validate our findings using the all atom molecular mechanics simulations. Strain transfer in a graphene bilayer structure was studied using the AIREBO potential^22^ for interatomic interactions using LAMMPS^23^. The results of the two approaches (Fig. S2, see SI) are in good agreement with each other and the critical strain values for slippage predicted from both approaches are very close (0.53% vs. 0.68%). Deviations observed for relatively large strains are due to the small size of the graphene bilayer, which is limited by the computational cost of the atomistic simulation.

### All the other heterostructures studied here show qualitatively same behavior as MoS_2_/WS_2_

The method developed here is general and in principle can be applied to study the strain transfer for any multilayer heterostructure as long as the interlayer potential is known. Most of the currently investigated vdW heterostructures have the same hexagonal symmetry as the MoS_2_/WS_2_ bilayer and hence the trends observed here will hold for all other heterostructures. To obtain a quantitative understanding in other heterostructures, we extend our calculations for a few other known vdW heterostructures (MoSe_2_/WSe_2_, MoS_2_/MoS_2_, WS_2_/WS_2_, MoSe_2_/WSe_2_). The magnitude of the maximum strain that can be transferred in a coherent manner and the shear-lag length are given in Table 1. For most of the known heterostructures, the shear-lag length ranges from 1.5 nm to 4.1 nm and the magnitude of the maximally transferred strain is in the range from 0.4% to 1.8%. For all of the structures except the bilayer graphene, BA stacking has higher energies compared to AB stacking and hence all commensurate domains have the AB stacking.

As shown in Fig. 5, the size of commensurate domains of BA and AB stackings is decreased under an increasing strain in the bottom layer. Changes in the stacking configuration are expected to have a significant effect on the optoelectronic performances of devices based on such stacked 2D heterostructures^24^. It has been shown earlier that the electronic and the quantum transport properties of vertically stacked vdW structures strongly depend on the stacking configurations. For example, Bao *et al*. have demonstrated^12^ that ABA-stacked trilayer graphene is semi-metallic while ABC-stacked trilayer graphene exhibits a semiconducting behavior with a tunable band gap. Similarly, for the transition metal dichalconides-based vdW heterostructures, it has been found that the polarization selection and brightness of the exciton emission show^25^ a strong dependence on the stacking order between two layers. Since our method shows that the spread of the commensurate-incommensurate domains depends on the strain in the substrate layer, it will be helpful in understanding the optoelectronic response of vdW heterostructure based flexible devices.

## Conclusions

A non-linear shear-lag model to study the strain transfer between different layers of 2D heterostructure with hexagonal symmetry has been developed.

For small-applied strains, the top layer is in a commensurate state with the bottom layer to minimize the vdW energy. Beyond a critical strain, a best compromise between the vdW energy and elastic energy is obtained when incommensurate-commensurate domains coexist.

Critical strain for slippage, maximum limit of the strain-transfer and sizes of the commensurate and incommensurate domains are predicated from the interlayer potential and elastic constants for a number of 2D heterostructures.

Due to the symmetry of the potential, commensurate domains are arranged in alternating sequences of AB/BA stacking, which have been experimentally observed recently.

## Additional Information

**How to cite this article**: Kumar, H. *et al*. Limits of Coherency and Strain Transfer in Flexible 2D van der Waals Heterostructures: Formation of Strain Solitons and Interlayer Debonding. *Sci. Rep.* **6**, 21516; doi: 10.1038/srep21516 (2016).

## Supplementary Material

Supplementary Information

## Figures and Tables

**Table 1:** Shear-lag length and critical strain to interfacial sliding for different heterostructures obtained using our continuum shear-lag model.

Heterostructure	Shear-lag Length λ (nm)	Critical Strain
MoS_2_-WS_2_	2.48	1.81%
MoSe_2_-WSe_2_	1.65	2.24%
GR-GR	4.11	0.53%
MoS_2_-MoS_2_	1.38	2.31%
WS_2_-WS_2_	2.28	1.90%
MoSe_2_-MoSe_2_	1.51	2.32%
WSe_2_-WSe_2_	1.52	2.56%
