# Supplementary material for: Limits of Coherency and Strain Transfer in Flexible 2D van der Waals Heterostructures: Formation of Strain Solitons and Interlayer Debonding
Source: Sci Rep. 2016 Feb 12;5:21516. doi: 10.1038/srep21516 (PMC4751462; doi:10.1038/srep21516)
Supplement: Supplementary Information [file srep21516-s1.pdf]

Supplementary Information

Limits of Coherency and Strain Transfer in Flexible 2D van der Waals Heterostructures: Formation of Strain Solitons and Interlayer Debonding

*Hemant Kumar, Liang Dong and Vivek B. Shenoy**[1]

Department of Materials Science and Engineering, University of Pennsylvania, Philadelphia, USA 19104

1. Calculation of the Interlayer Shear Potential

The interlayer shear energy surface is defined as the change in interlayer vdW energy as a function of the relative displacement between the primitive unit cells of the top and the bottom layers. For the heterostructures studied here, the PES has the hexagonal symmetry of underlying lattice and the most general functional form can be written as:

$$U_s = c_0(z) + \sum_m c_m(z) e^{\{i\vec{G}_m.\delta\vec{u}\}}, \quad \text{(Eq. S1)}$$

where $c_0(z)$ and $c_m(z)$ are coefficients that are dependent on the interlayer separation distance, $\vec{G}_m$ represents the reciprocal lattice vector of the underlying lattice, and δu and δv are the relative displacements from the minimum energy stacking (AB stacking) along the x-axis and y-axis, respectively. In an earlier study [1] , we show the summation in Eq. (S1) only needs to be done over the reciprocal vectors in the first Brillion zone. Simplifying Eq. (S1) with this summation leads to the following functional form characterized by three coefficients $c_0(z), c_1(z)\ and\ \phi$:

$$U_s = c_0(z) + 2c_1(z)\cos(\phi - G\delta v) + 4c_1(z)\cos\left(\frac{G\delta v}{2} + \phi\right)\cos\left(\frac{\sqrt{3}G\delta u}{2}\right), \quad \text{(Eq. S2)}$$

In Eq. (S2), G is equal to $4\pi/a\sqrt{3}$, where 'a' is the lattice constant. For small deformations, Taylor's expansions can be applied to the coefficients $c_0(z)$ and $c_1(z)$ around the minimum energy separation z_0 :

$$U_s = c_0(z_0) + c_0'(z_0)(z - z_0) + 2\{c_1(z_0) + c_1'(z_0)(z - z_0)\}\left\{\cos(\phi - G\delta v) + 2\cos\left(\frac{G\delta v}{2} + \right.\right.$$

[1] Corresponding Author: vshenoy@seas.upenn.edu

$\phi\Big)\cos\left(\frac{\sqrt{3}G\delta u}{2}\right)\Big\}$. (Eq. S3)

The values of $c_0(z_0), c_1(z_0)$ and ϕ are fitted from Eq. (S3) using the energy of the bilayer/heterostructure for different stacking configurations that were obtained by displacing the unit cell of the top layer with respect to that of the bottom layer in the *xy*-plane. To compute the energy, we use first principles calculations based on density functional theory (DFT) as implemented in the Vienna ab-initio simulation (VASP) code [2]. DFT calculations were performed using Perdew–Burke–Ernzerhof (PBE) functional of the generalized gradient approximations (GGA) [3]. The vdW interactions were treated using the DFT-TS method [4]. Having obtained these coefficients, we plot the PES according to Eq. (S3) as shown in Fig. 2 of the manuscript.

2. *1D Shear-lag Model*

For 1D calculations, we constrain the projection of interlayer shear potential along the zigzag direction, which is obtained by setting all displacement along the *y* direction to be zero, *i.e.*, $\delta v = 0$. Therefore, Eq. (S3) is simplified as:

$$U_s = c_0(z_0) + c_0'(z_0)(z - z_0) + 2cos\phi\{c_1(z_0) + c_1'(z_0)(z - z_0)\}\left\{1 + 2\cos\left(\frac{2\pi\delta u}{a}\right)\right\} \quad \text{(Eq. S4)}$$

Elastic energy per unit area of the two layers for a given strain is:

$$U_{el} = E_b\left(\frac{du_b}{dx}\right)^2 + E_t\left(\frac{du_t}{dx}\right)^2 + \kappa_t\left(\frac{d^2w_t}{dx^2}\right)^2, \quad \text{(Eq. S6)}$$

where u_t , u_b are the displacements along the applied strain in top and bottom layer, respectively and w_t is out-of-plane displacements of the top plate. (For simplicity, we assume that only the top layer has an out-of-plane displacement and hence, $z - z_0 = w_t$.) Now, the total energy (Elastic + Shear) per unit length will be:

$$U_{tot} = \int_0^L dx\left[E_b\left(\frac{du_b}{dx}\right)^2 + E_t\left(\frac{du_t}{dx}\right)^2 + \kappa_t\left(\frac{d^2w_t}{dx^2}\right)^2 + c_0 + c_0'w_t + 2cos\phi\{c_1 + c_1'w_t\}\left\{1 + 2\cos\left(\frac{2\pi\delta u}{a}\right)\right\}\right]. \quad \text{(Eq. S7)}$$

For brevity, we have removed explicit expression of the interlayer distance dependence of c_0 and c_1. Variational minimization of the total energy in Eq. (S7) with respect to the variations of u_b, u_t *and* w_t

leads to the following differential equations:

$$\frac{d^2u_t}{dx^2} - \frac{4\pi cos\phi(c_1+c_1'w_t)}{aE_t}\sin\left(\frac{2\pi\delta u}{a}\right) = 0,$$

$$\frac{d^2u_b}{dx^2} + \frac{4\pi cos\phi(c_1+c_1'w_t)}{aE_b}\sin\left(\frac{2\pi\delta u}{a}\right) = 0,$$

$$\frac{d^4w_t}{dx^4} - \frac{4cos\phi c_1'}{\kappa_t}\sin^2\left(\frac{\pi\delta u}{a}\right) = 0, \qquad \text{(Eq. S8)}$$

These coupled differential equations were solved numerically using the finite-element method with the boundary conditions $u_b(0) = u_t(0) = 0$, $u_t'(L) = 0$ (the top layer has free ends), and $u_b(L) = l$ (the bottom layer has an applied strain with a given displacement at the edges) where l is the applied displacements at the boundary to create a strain in the bottom layer. Numerical solutions of Eq. (S8) are presented in Figure S1.

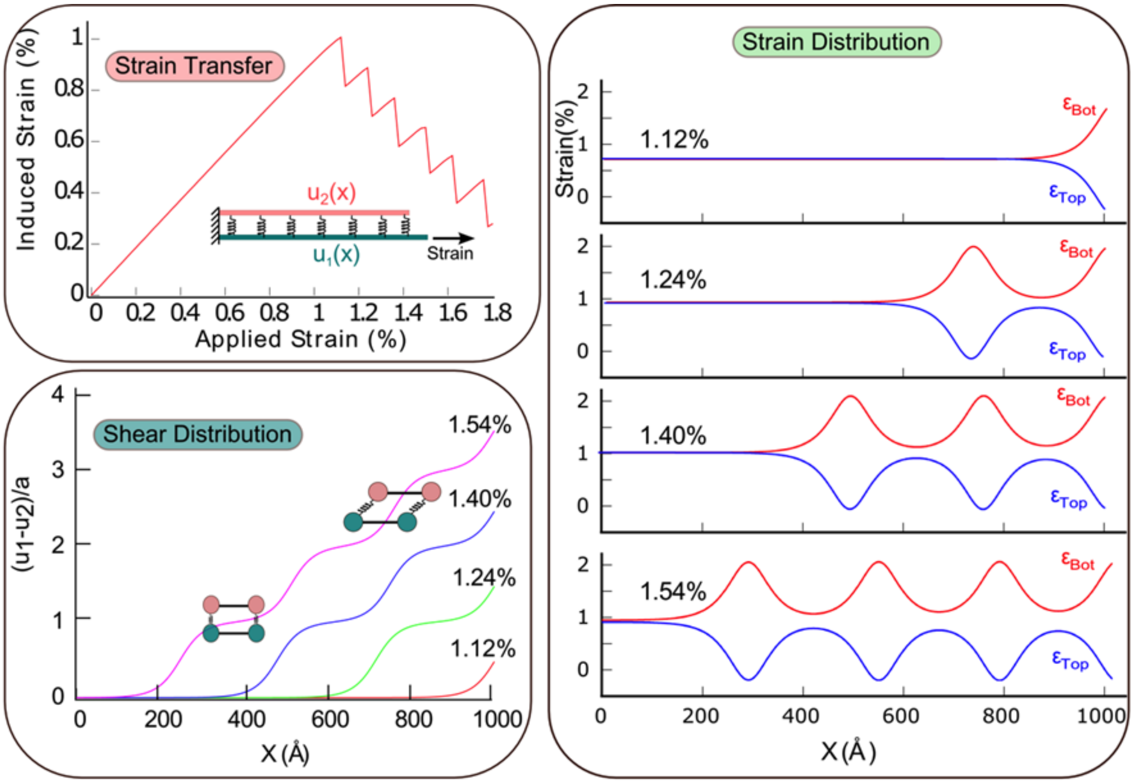

FIG. S1. *Results from the 1D shear-lag model for the graphene bilayer. A) Average strain in the top layer as a function of the applied strain in the bottom layer. Both layers are in commensurate phase for the applied strain from 0 to ~1.1%, wherein strain transferred to the top layer increases linearly with the applied strain. Beyond the critical applied strain of 1.1%, the incommensurate domains appear and hence the average strain in the top layer starts decreasing with increasing applied strain. Each dip for*

applied strains >1.1% correspond to the occurrence of an incommensurate domain. B) Distribution of the interlayer shear along the X axis under different values of the average strain (1.12%, 1.24%, 1.40%, and 1.54%) in the bottom layer. For the curve under a given strain (e.g., 1.54%), the integer terraces correspond to the commensurate domains while the climbing wall between two terraces correspond to the incommensurate domains. C) The strain distribution in both layers along X for different values of applied strains.

3. *Analytical Solutions for 1D model*

To gain further insight, we also obtain the analytical solutions with some approximations. Numerical solutions show that strain-transfer is linear until first incommensurate domain. Hence, for small strains, the sinusoidal potential can be approximated with the harmonic potential. Furthermore, bending rigidity of 2D materials is orders of magnitude smaller than the elastic modulus and hence the energy cost of bending can be ignored. With the approximation $sin\theta = \theta$ and ignoring the energy cost of the bending, Eq. (S8) reduce to:

$$\frac{d^2u_t}{dx^2} - \frac{8\pi^2 cos\phi c_1}{aE_t}\left(\frac{\delta u}{a}\right) = 0, \quad \text{(Eq. S9)}$$

$$\frac{d^2u_b}{dx^2} + \frac{8\pi^2 cos\phi c_1}{aE_b}\left(\frac{\delta u}{a}\right) = 0, \quad \text{(Eq. S10)}$$

Subtracting Eq. (S9) with Eq. (S10) gives the interlayer shear:

$$\frac{d^2\delta u}{dx^2} + \frac{8\pi^2 cos\phi c_1}{a^2}\left(\frac{E_b+E_t}{E_bE_t}\right)\delta u = 0, \quad \text{(Eq. S11)}$$

where $\delta u = u_b - u_t$. Replacing $(a^2E_{eff})/(8\pi^2c_1cos\phi) = -\lambda^2$ where $E_{eff} = E_bE_t/(E_b + E_t)$, we get :

$$\frac{d^2\delta u}{dx^2} = \frac{\delta u}{\lambda^2}, \quad \text{(Eq. S12)}$$

and similarly, adding Eq. (S9) and Eq. (S10) leads to:

$$\frac{d^2(u_b+u_t)}{dx^2} = 0. \quad \text{(Eq. S13)}$$

The boundary conditions $u_b(0)=0$ and $u_t(0)=0$ give the following solutions:

$$u_t = \frac{Ax}{2} - Bsinh\left(\frac{x}{\lambda}\right), \quad \text{(Eq. S14)}$$

$$u_b = \frac{Ax}{2} + Bsinh\left(\frac{x}{\lambda}\right), \quad \text{(Eq. S15)}$$

A and B in Eqs. (S14) and (S15) are can be computed from the boundary conditions $u'_t(L)=0$ and $u_b'(L) = \epsilon$ so that:

$$A = \epsilon, \tag{Eq. S16}$$

$$B = \frac{\lambda\epsilon}{\cosh(L/\lambda)}, \tag{Eq. S17}$$

Substituting these value in Eq. (S14) and Eq. (S15) gives:

$$\delta u = \frac{\lambda\epsilon \sinh\left(\frac{x}{\lambda}\right)}{\cosh\left(\frac{L}{\lambda}\right)}, \tag{Eq. S18}$$

As it is clear from this expression, interlayer shear δu will decay over a length scale λ which is know as shear-lag length. Eq. (S18) can also be used to predict the critical strain of slippage. As shown in the numerical results, when the interlayer shear at the edge equals half of the lattice constant $(i.e. \delta u = a/2)$, slippage begins to occur. Hence, the critical applied strain at the edge to begin slip is:

$$\epsilon_c = \frac{a}{2\lambda} \coth\left(\frac{L}{\lambda}\right) \tag{Eq. S19}$$

4. *Comparison with Atomistic Simulations*

To validate our method, we compare strain-transfer response from our model with all atom molecular mechanics simulations for the graphene-bilayer system. We simulated a graphene bilayer sheet (100 nm by 20 nm) using the AIREBO potential [5] using LAMMPS [6] software package. Default value of the Lennard-Jones (LJ) parameters in the AIREBO potential give very small value of the interlayer shear-modulus as compared to the experiments but modified values of LJ parameters ($\epsilon_c = 0.04544\ eV\ and\ \sigma_c = 0.34\ nm$) have been shown to reproduce [7] experimental shear-modulus of graphene. Strain-transfer values obtained from MD simulations have been shown in the Figure below.

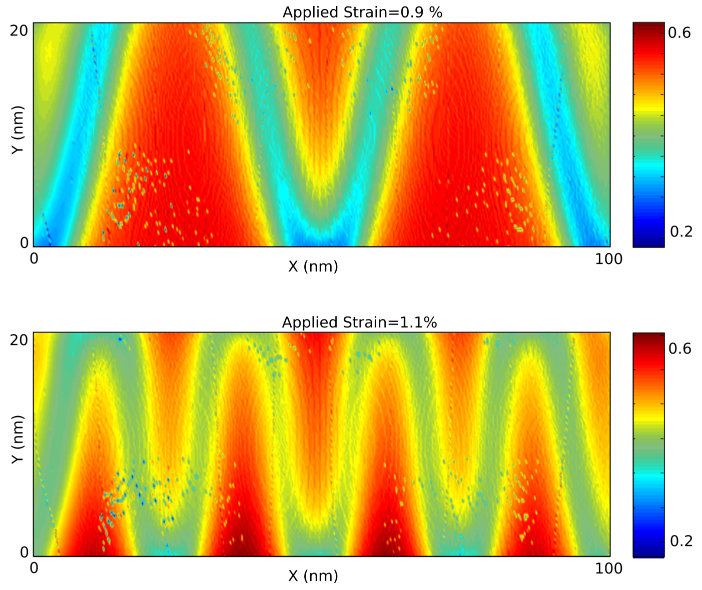

Figure S2: Strain transferred in the top layer in a graphene bilayer structure was studied using AIREBO potential (with modified interlayer vdW interaction). The results of the two approaches are in good agreement with each other and the critical strain values for slippage predicted from both approaches are very close (0.53% vs. 0.68%). Deviations observed for relatively large strains are due to the small size of the graphene bilayer, which is limited by the computational cost of the atomistic simulations.

References:

[1] H. Kumar, D. Er, L. Dong, J. Li, and V. B. Shenoy, Sci. Rep. **5**, 10872 (2015).

[2] G. Kresse and J. Furthmüller, Phys. Rev. B **54**, 11169 (1996).

[3] J. P. Perdew, K. Burke, and M. Ernzerhof, Phys. Rev. Lett. **77**, 3865 (1996).

[4] A. Tkatchenko and M. Scheffler, Phys. Rev. Lett. **102**, 73005 (2009).

[5] S. J. Stuart, A. B. Tutein, and J. A. Harrison, J. Chem. Phys. **112**, 6472 (2000).

[6] S. Plimpton, J. Comput. Phys. **117**, 1 (1995).

[7] Y. Shen and H. Wu, Appl. Phys. Lett. **100**, 101909 (2012).
